# Peripheral Manifestations in Age Related Macular Degeneration: A Review of Imaging and Findings

**DOI:** 10.3390/jcm10173993

**Published:** 2021-09-03

**Authors:** Andrew Pivovar, Patrick Oellers

**Affiliations:** 1 Department of Ophthalmology and Visual Sciences, Upstate Medical University, Syracuse, NY 13202, USA; pivovara@upstate.edu; 2 Retina-Vitreous Surgeons of Central New York, Liverpool, NY 13088, USA

**Keywords:** age-related macular degeneration, peripheral, ultra-widefield, fluorescein autofluorescence, grid analysis

## Abstract

Purpose: To review novel findings in research with ultra-widefield imaging for analysis of peripheral manifestations in macular degeneration (AMD). We introduce the evolving widefield imaging modalities while summarizing the analytical techniques used in data collection of peripheral retinal findings thus far. Our review provides a summary of advancements to date and a commentary on future direction for AMD research. Methods: This is a literature review of all significant publications focused on the relationship between AMD and the retinal periphery conducted within the last two decades. Results and Conclusion: Promising research has been undertaken to elucidate peripheral retinal manifestations in macular degeneration using novel methodology. Advancements in ultra-widefield imaging and fundus autofluorescence have allowed us to elucidate peripheral retinal pigmentary changes, drusen deposition, and much more. Novel grid overlay techniques have been introduced to aid in analyzing these changes for pattern recognition and grouping of findings. This review discusses these findings in detail, providing evidence for the pan-retinal manifestations of AMD. Inter-study discordance in analytical approach highlights a need for more systematic future study.

## 1. Introduction

Age-related macular degeneration (AMD) is the leading cause of irreversible blindness in the elderly population of developed countries worldwide. Presenting with a variety of manifestations, some signs of AMD include drusen deposition below the retinal pigment epithelium and geographic atrophy (GA). AMD is categorized into the exudative (wet) and non-exudative (dry) forms. The presence of choroidal neovascularization in exudative AMD differentiates this disease from the dry, usually more slowly progressing, form [1]. While much research has been done to understand the pathophysiology of macular manifestations in AMD, the periphery of the retina needs closer study. While studies show that peripheral pigmentary, drusenoid, and atrophic retinal changes are indeed common in AMD patients, the impact of these findings on the disease is not well understood. Recent developments in imaging technology have allowed us to analyze peripheral retinal findings more systematically. With the growing application of ultra-widefield (UWF) fundus imaging for detection of peripheral retinal manifestations, we have recently developed a novel UWF image-based grid grading system for methodical study of peripheral retinal findings [2]. In this review, we present recent studies and technological advancements made in identifying the peripheral manifestations of AMD in hope of developing a more cohesive understanding of the disease. We aim to give a background on widefield retinal imaging and image analysis while sequentially describing the recorded findings of AMD studies.

## 2. Available Cameras

Fundus imaging has steadily evolved from the first fundus cameras to allow physicians to capture up to 220°, or 97% of the retina [3]. The onset of a new era of widefield imaging has broadened our horizon in studying the retinal periphery. While use of this technology has steadily increased, a consensus on the definition of widefield imaging has yet to be established. Recently, an international panel of retinal imaging specialists have ascribed the term widefield to images depicting the area beyond the posterior pole yet posterior to the vortex vein ampulla in all four quadrants [4]. Concordantly, the panel has defined ultra-widefield images as including the retina anterior to the vortex vein ampullae stretching to the pars plana in all four quadrants [4]. A variety of UWF imaging cameras are clinically available for image acquisition.

One such apparatus is the Optos UWF camera (various models, Optos plc, Dunfermline, UK). This confocal scanning laser ophthalmoscope (cSLO) allows for imaging of up to 200° of the fundus without dilation in many instances [3]. The Optos automontage function potentially expands this capability to allow for 220° of fundus imaging by combining four gaze steered images. Additionally, Optos cameras are capable of performing fluorescein angiography (FA), indocyanine green angiography (ICGA), fundus autofluorescence (FAF), and, more recently, also optical coherence tomography (OCT) [3]. A systematic review of widefield-related articles has found the Optos camera as one of the most essential tools for identifying vascular pathology and peripheral retinal disorders [5]. This widely used camera is not without its limitations; peripheral distortion and eyelash overlay are prevalent in imaging. The lack of true color with potential for various artifacts is frequently critiqued [3].

The Clarus 500 camera (Carl Zeiss, Meditec AG, Jena, Germany) captures up to 135° in a single image; however, combined images from different angles can improve the view to 200° [6]. In contrast to the Optos system, Clarus cameras provide true color images. While the Clarus 500 may produce a slightly narrower viewing angle, it may prove more effective for true color retinal imaging [3]. The Clarus 700 system provides FAF, FA, and ICGA capabilities.

The Heidelberg Widefield Module, or Heidelberg Spectralis cSLO (Heidelberg Engineering, Inc., Heidelberg, Germany) allows for a 55° view in noncontact lens format and 105° if performed with a contact lens [3]. In addition to providing a generally more limited widefield view, the Heidelberg camera’s limitations in visualizing the superior and inferior periphery are most pronounced.

The Staurenghi Lens System (Ocular Staurenghi 230 SLO Retina Lens; Ocular Instruments Inc, Bellevue, Washington) uses a contact lens module that allows for a 150° view of the retina via cSLO and a convex-concave lens system [3]. However, this method of UWF image acquisition requires topical anesthesia and skilled operation of the contact lens.

## 3. Autofluorescence

The basis of FAF imaging rests upon the fluorescent nature of lipofuscin to allow the study of the topography and physiology of the retinal pigment epithelium [7]. Lipofuscin accumulates as a byproduct of incomplete degradation of lipids in phagocytosed photoreceptor outer segments within RPE cells [7]. Excessive deposition of lipofuscin in RPE cell lysosomes is a hallmark of the aging process in the eye. The molecules that compose lipofuscin, such as N-retinyl-N-retinylidene ethanolamine (A2E), are not recognized by lysosomal enzymes and thus cannot be efficiently degraded. Many studies have shown that above-threshold lipofuscin accumulation causes RPE cell apoptosis and contributes to age-related vision loss [7]. The natural FAF autofluorescence pattern of the eye is characterized by low intensity or dark pixel values. High intensity autofluorescence visualized as light pixel values is a manifestation of increased lipofuscin in RPE cells and lipid-rich drusen deposits seen in AMD. Lower than normal autofluorescence values can be seen in geographic atrophy of the retina, where RPE-derived lipofuscin granules are absent. Thus, areas of increased metabolic stress appear bright while areas of RPE atrophy appear darker than the background [7]. FAF imaging is highly effective for visualizing drusen deposition in AMD and monitoring for progression of geographic atrophy.

The newly developed ultra-widefield cameras have used confocal scanning ophthalmoscopy to push the boundaries of what we can visualize with autofluorescence imaging of the fundus [8]. Before the development of cSLO systems, standard fundus camera photography acted as our primary mode of FAF image acquisition, limiting our view of the peripheral retina and ability to study the pathogenesis of diseases such as AMD. The application of FAF in widefield cSLO systems has elevated our understanding of peripheral manifestations in AMD.

## 4. Early Widefield Studies

Since the mid-2000s, an effort has been made to understand and characterize peripheral retinal changes in AMD. Early studies have found peripheral pigmentary changes in AMD to be associated with Complement Factor H polymorphisms, suggesting a high-risk genotype linked to peripheral manifestations [9,10,11]. More specifically, the CFHY402H genotype has been found to be associated with peripheral drusen and the CFHrs1410996 genotype with reticular pigment [10]. Other studies have attempted to delineate the differing morphology of drusen in the macula and retinal periphery by examining drusen ultrastructure and composition [12]. Dividing the types of drusen into hard, soft, and compound, Rudolf et al. found significant differences in drusen type between the macula and the periphery. Soft drusen with abundant basal laminar deposits were found exclusively in the macula, while compound drusen were found in the periphery. Hard drusen were found to be the most abundant type, seen throughout the retina [12]. The presence of structurally dissimilar extramacular drusen has warranted further comparison of peripheral and central retinal findings in AMD.

With the growing interest in peripheral findings, FAF and pseudocolor imaging were among the primary methods used to identify peripheral retinal abnormalities in AMD patients. Retrospective studies of FAF changes in AMD patients have demonstrated an increased prevalence of peripheral autofluorescence abnormalities [13,14,15,16]. For example, Reznicek et al. found that FAF imaging produces greater peripheral and central signal in AMD patients when compared to healthy controls [13]. Heussen et al. showed a higher prevalence of abnormal atrophic, hemorrhagic, and drusenoid peripheral autofluorescence patterns in AMD patients compared to control (70% vs. 18%) [14].

Around the same time, granular, patchy, and reticular autofluorescent changes were identified by widefield imaging in a 2012 retrospective study of AMD patients by Witmer et al. [15]. Shortly afterwards, Tan et al. described granular, mottled and nummular FAF abnormalities that occurred more frequently in exudative compared to non-exudative AMD and healthy eyes (86% vs. 72.8% vs. 18.4%) in their study cohort [16]. In studying different wet AMD subtypes, Suetsugu et al. corroborated these findings, depicting that the eyes of Japanese patients with exudative AMD had a higher prevalence of abnormal mottled, granular, and nummular FAF findings [17].

An Age-Related Disease Study 2 (AREDS 2) report comparing peripheral retinal changes in 484 participants with AMD to healthy controls has found additional evidence to solidify our understanding of AMD as more than a macular condition [18]. The study found drusen to be more prevalent in the mid and far periphery of AMD eyes when compared to the control. However, pigmentary changes associated with more advanced AMD were much less prevalent in the periphery when compared to the posterior pole [18].

**Figure 1:**
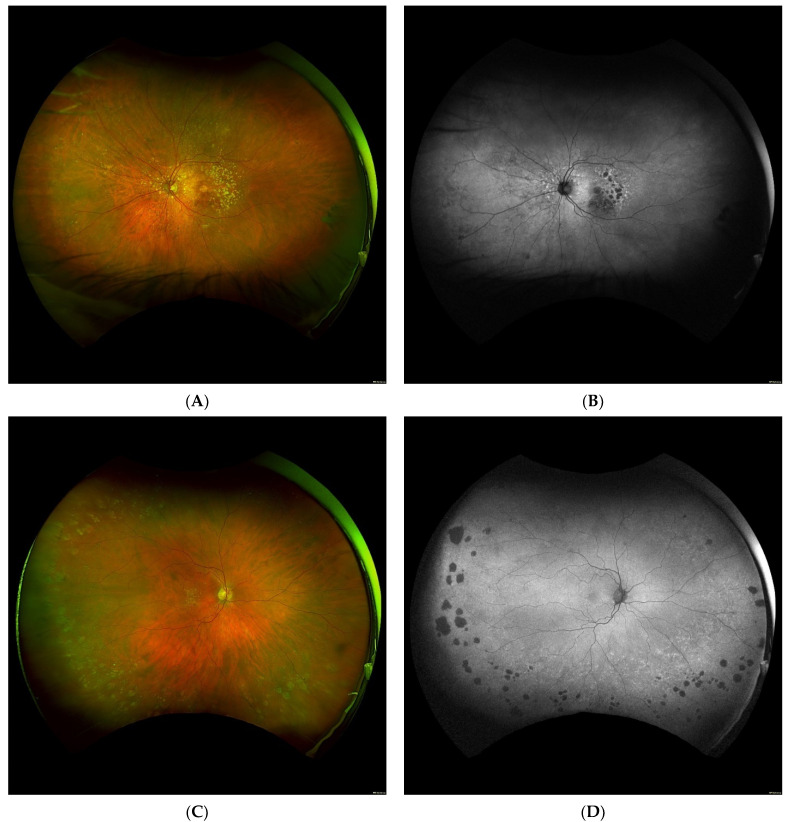

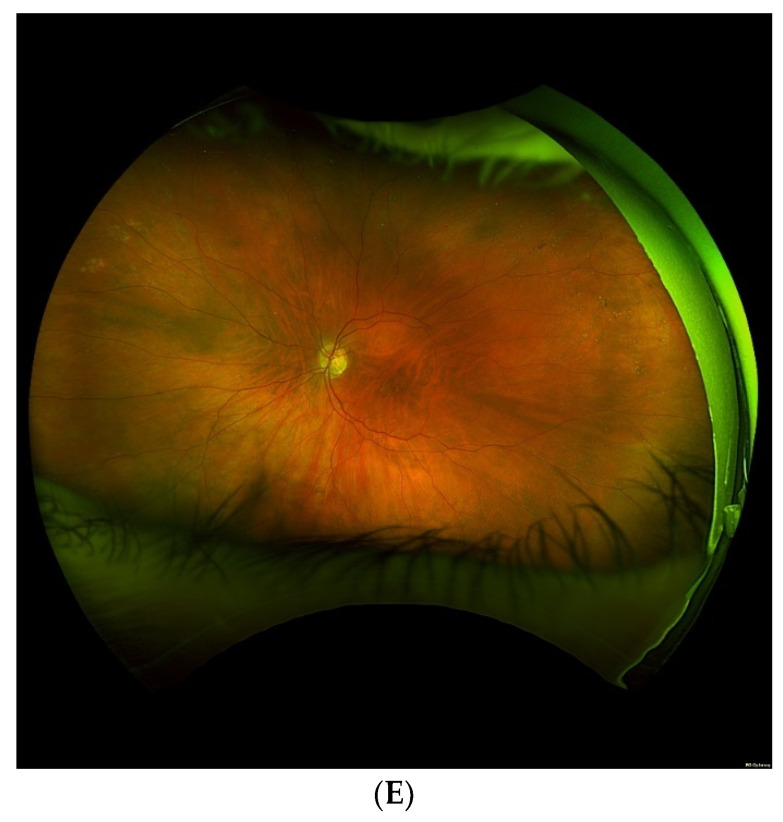
Exemplary ultra-widefield images of eyes with macular degeneration and peripheral abnormalities. Pseudocolor and corresponding autofluorescence of a left eye demonstrating multifocal retinal pigment epithelium (RPE) atrophy and drusen in the macula with peripheral drusen and multifocal RPE atrophy, especially nasal to the optic nerve. Note there is a glaucomatous disk hemorrhage and a peripheral retinal tear status post laser retinopexy (**A**,**B**). Another example demonstrates a right eye with macular drusen and peripheral drusen and multifocal atrophy in the far periphery (**C**,**D**). A left eye with macular drusen and pigment changes and drusenoid bodies and mild reticular pseudopigmentation in the periphery. Note eyelash artifact inferior (**E**).

As seen in these studies, there is a plethora of evidence to suggest an increased prevalence of peripheral retinal changes in AMD patients in comparison to the healthy controls. Figure 1 demonstrates a few examples of patient with macular degeneration and peripheral retinal findings. While our understanding of AMD as an extra-macular disease continues to grow, the methods employed to characterize these peripheral findings have proven to be somewhat subjective and discordant. In particular, documented approaches to grid analysis of the retinal periphery in AMD patients have seen much incongruity.

## 5. Proposed Grid Systems

In the following section, we present a chronological analysis of the grids used in study of Optos-based images of the retinal periphery. Reznicek et al. were among the first to conduct grid analysis of UWF FAF images, using the Optos 200Tx. The group utilized a 7-ring grid totaling 48 zones of optic disk-length width [13]. FAF measurements were taken in four of the peripheral areas (34, 35, 38, and 39). These four segments encompassed minor areas of the nasal and temporal retinal periphery.

In comparing the frequency of peripheral retinal abnormalities in FAF versus pseudocolor images, Heussen et al. graded the lesions largely by superior, inferior, temporal, and nasal segmentation [14]. In a subsequent study, Tan et al. defined the peripheral zone as anything outside the central 30 degrees centered on the fovea [16]. For their FAF analysis, the Tan group adopted the same four quadrant analysis as before, attempting a crude documentation of peripheral abnormalities by clock hour involvement [16].

Nomura et al. defined the midperipheral zone around the macula by two circles of different diameter using multiples of papilla diameter [19]. The midperiphery was defined as the area between the 3 papilla diameter and 9 papilla diameter circles [19]. As evident by the discordance of approaches to grading lesions of the peripheral retina, a systematic approach to grading is yet to be established.

In 2015, Lengyel et al. added to the growing number of UWF studies with the Optos 200Tx [20]. The authors divided the retina into five zones, with zones 1–3 based on the original definition of the standard macular grid as established by the international classification and age-related eye disease systems [21,22]. Zones 4 (mid periphery) and 5 (far periphery) were arbitrarily chosen without correction for peripheral distortion. These two zones were further subdivided into four quadrants for additional analysis. While this study depicted a possible method by which to grade peripheral lesions, it is limited by neglecting to correct for distortion.

In evaluating the presence of abnormal FAF patterns in AMD eyes, Suetsugu et al. centered their grid on the macula, covering the central 30 degrees while splitting the retinal periphery into four 90 degree quadrants: superior, inferior, nasal, and temporal [17]. This approach is similar to that of Tan and Heussen [14,16]. This method of separation cannot be called equivalent, however, as Tan’s center covered much more of the optic disk than Suetsugu’s. A subsequent study of peripheral FAF findings by Guduru et al. also neglected to add more than a four quadrant separation of the retina into the 90 degree quadrants discussed previously [23]. The evident limitation lies in the simplicity of these grids, isolating the data from more meticulous analysis.

Up until now, all studies mentioned have chosen to center their grids on the fovea. In the AREDS 2 report on peripheral findings in AMD patients, the chosen grid was centered instead on a midpoint between the optic disk and fovea [18]. No explicit reason was given for this change. The 3-zone grid was adopted from a 1992 Study of Ocular Complications of AIDS, analyzing cytomegalovirus retinitis manifestations in the periphery [24]. Zone 1 had a radius of 3 optic disk diameters, corresponding to the posterior pole of the retina. Zone 2 was defined by the area between 9 and 3 disk diameters, while zone 3 the region anterior to zone 2. Zones 1 and 2 were further divided into four quadrants (superonasal, superotemporal, inferonasal, inferotemporal) and zone 3 was subdivided into superior and inferior hemispheres [18].

In their 2017 study, Bae et al. took a different approach by studying ultra-widefield fluorescein angiography of the retinal periphery [25]. To analyze peripheral reticular pigmentary degeneration, the group divided the retina into four quadrants, using hour units to measure the extent of the lesions.

A 2020 study of peripheral UWF FAF findings in AMD by Küçükiba et al. used fovea-centered images with a three-zone grid based upon the grids of the AREDS2, Nomura, and Tan studies [14,16,18,26]. Zones 1 and 2 were both quantified at 3 and 9 optic disk diameters respectively, with zone 3 as anything peripheral to the other two. This provides a sense of continuity and comparability between the four studies. To refine the previous grids, the group divided all three zones into four quadrants, not just the first two as seen in the AREDS2 study [18,26].

The proposed grid systems used in analysis of peripheral FAF manifestations in AMD can be divided into four quadrant and three-zone models. While the three-zone grid provides a more detailed interpretation of peripheral lesions, more can be done to develop a more standardized and accurate grid. The difference in autofluorescence findings among the four quadrants and various zones is a relevant factor for developing a better grid system. For instance, studies have shown the nasal quadrant has more frequent involvement in peripheral findings [16,23]. This finding, in combination with the multitude of proposed models, stresses the importance of developing more standardized methods for grading lesions with better reproducibility and accuracy.

## 6. Boston Grid

In order to develop a standardized method to characterize peripheral retinal changes, we have demonstrated a novel grid approach in our cross-sectional observational case series [2]. With the focus on ultra-widefield (UWF) imaging to diagnose peripheral manifestations, we have presented a method by which UWF imaging may be used to define these changes while accounting for peripheral distortion.

**Figure 2:**
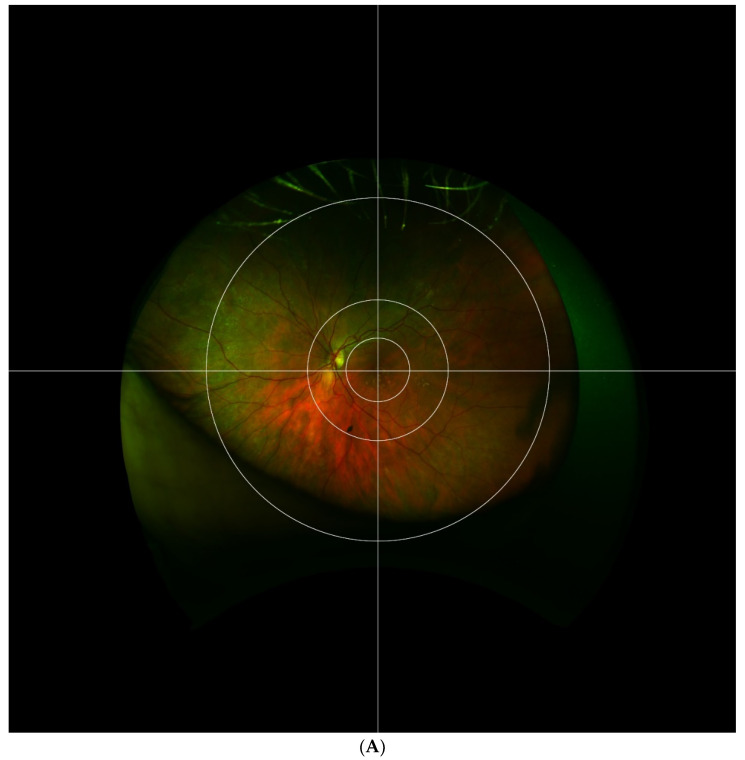

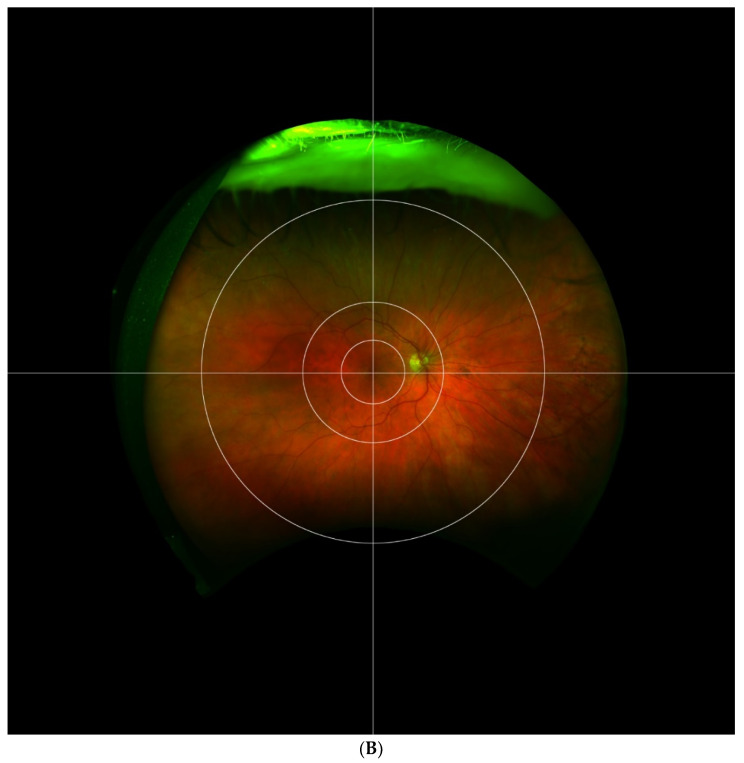
The Boston grid is depicted superimposed on fundus images of right (**A**) and left (**B**) eyes. The grid is composed of 3 concentric circles centered on the fovea, separating the retina into central, perimacular, mid-peripheral, and far-peripheral areas. Horizontal and vertical lines further separate the retina into superior, inferior, temporal, and nasal quadrants, producing a total of 12 zones.

**Figure 3:**
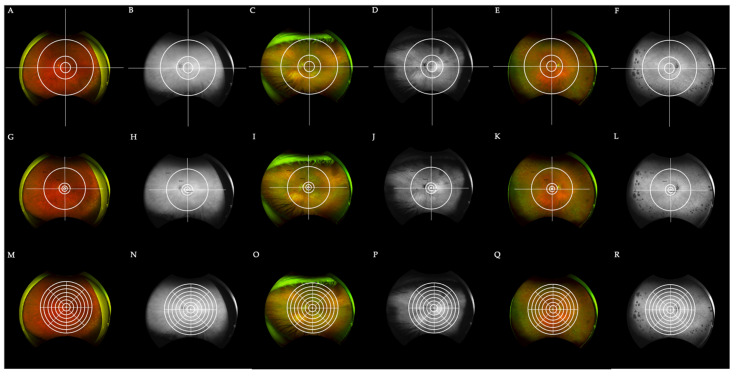
A comparison of the Boston grid to two other discussed grids for a total of 3 patients with AMD (**A**–**F**). Fundus pseudocolor and autofluorescence imaging of 3 patients diagnosed with AMD with the Boston grid overlay. Fundus imaging of the first patient is depicted in (**A**,**B**,**G**,**H**,**M**,**N**). Peri-macular drusen in (**C**) is best captured by the Boston grid when compared with the Lengyel (**I**) and Reznicek (**O**) grids. Similarly, central drusen in (**E**) is best circumscribed by the Boston grid. (**G**–**L**) The exact same photos as in (**A**–**F**) with the recreated grid by Lengyel et al. superimposed. In comparison to the Boston grid, the Lengyel grid lacks comprehensive peri-macular and mid-peripheral zones, with zones 1–3 found strictly in the macula and zone 4 encompassing both the mid-periphery and peri-macular area. (**M**–**R**) An overlay of the recreated Reznicek et al. grid on the images presented previously. The 48 partitions seen here do not clearly distinguish the mid-periphery from the far-periphery and peri-macular areas of the retina, but create much further detail which might be helpful in select cases. For instance, the peri-macular drusen in (**O**) can be found in approximately 12 zones as opposed to one or few distinct zones. Grids were recreated based on instructions detailed elsewhere [13,20].

The Boston grid proposed in our 2017 study is composed of three concentric circles with a crosshair centered on the fovea as depicted in Figure 2. The first circle encompasses the macula within the vascular arcades, measuring 29 mm. This zone is adapted from the Early Treatment of Diabetic Retinopathy Study (ETDRS) grid which was used by the Age-related Eye Disease Study (AREDS) [22]. The second circle encompasses the perimacular retina and temporal arcades at a radius of 65 mm. This division adds an extra zone around the central macula, one that has shown a high prevalence of retinal pathology while remaining peripheral to the center. The third circle separates the mid periphery from the far periphery at a radius of 160 mm. This division was chosen according to the location of the vortex veins. The crosshair grid further splits the retina into four quadrants: superonasal, inferonasal, superotemporal, and inferotemporal. In total, the grid divides the extramacular retina into 12 zones centered at the macula, not the optic nerve. This allows for the temporal mid-periphery to be more analogous to the nasal mid-periphery. A comparison of the Boston grid to the Lengyel [20] and Reznicek [13] grids discussed previously is made in Figure 3. Based upon the pseudocolor and autofluorescence fundus photography of three separate eyes with AMD, the Boston grid may provide the most clear and effective way to distinguish the perimacular area from the mid and far periphery of the retina.

Using new proprietary Optos software, UWF images were also corrected for peripheral vision distortion seen in image acquisition, allowing for greater accuracy in measurement of peripheral lesions with the new grid. Both the ellipsoid mirror used by Optos cameras to capture images and the natural curvature of the retina lead to a peripheral distortion of images captured by ophthalmoscopes. A novel image transformation software provided by Optos allowed us to correct for peripheral distortion to improve the accuracy and grading of abnormalities. The Boston grid works synchronously with this new software to eliminate grading error. While studies prior to 2017 have not employed such corrective software, image distortion is now integrated in new generation cameras. With the added perimacular zone and correction for vision distortion, the Boston grid may present significant advantages over previously described analytic methods.

Our new method for grading peripheral retinal changes is not without limitations. For one, the Optos camera used in acquiring UWF images with extensive peripheral visualization is prone to producing artifacts leading to possible misinterpretation of findings. Thus, UWF images should be interpreted by experienced graders to avoid misinterpretation. The grid is also limited in its applicability to subjects with up to −6D refractive error due to increase in fundus size associated with myopia. Despite the minor drawbacks, our opinion is that the Boston grid lays the proper framework for future study of peripheral retinal changes by remaining true to retinal dimensions and peripheral geometric distortion in UWF images.

## 7. Recent Studies

With the development of various UWF imaging modalities, we can begin to analyze the effect that extramacular manifestations have on AMD pathogenesis and symptoms. There is concrete evidence to suggest the implication of peripheral retinal lesions in AMD and retinal disease as a whole [27]. The most recent studies have helped us to understand the clinical significance of these peripheral lesions, setting the scene for application of our novel grid paradigm. Concurrently, research is beginning to elucidate a connection between tangible symptoms and peripheral retinal changes.

The results of a prospective cross-sectional study by Laíns et al. support an association between dark adaptation and peripheral retinal pigmentary change in the retina of AMD afflicted persons [28]. At total of 128 eyes (75% AMD and 25% control) were included in this study. For UWF image processing, Laíns et al. utilized our proposed fundus grid to analyze and classify peripheral pathology in AMD eyes. This grid allowed for clear analysis of drusen deposits and reticular pigmentary changes in the mid-peripheral and far-peripheral zones. Based on pseudocolor photos of the mid and far-periphery, peripheral reticular pigmentary changes were found to be associated with delayed rod intercept times (dark adaptation). Of note, reticular pigmentary changes were found only in AMD patients and not in controls in this study. Furthermore, with use of the classification system for FAF abnormalities proposed by Tan et al., [16] mottled decreases in FAF pattern in the midperiphery were also found to be significantly associated with delayed rod intercept times. Interestingly, there was no statistically significant association between peripheral drusenoid changes and rod intercept times.

Here again, UWF analysis is critical in developing a clear understanding of peripheral retinal manifestations and pathology. With the help of the Boston grid, Laíns et al. have succeeded in developing a new avenue of research into the interplay between functional vision and peripheral findings in AMD. This research supports the notion that AMD is not just a macular disease but a pathology with important peripheral manifestations that play an impactful role and deserve further study.

Closer study is beginning to unveil even more implications of altered peripheral retinal function in development of AMD pathology. A 2019 study by Tsunoda et al. lends more evidence to support a possible association between late-onset night blindness and a trickling subtype of AMD [29]. As described by Holz et al., trickling type AMD is a rapidly progressive phenotype of geographic atrophy characterized by trickling pattern of hypo-autofluorescence in FAF images [30]. Using ophthalmoscopy, FAF, and full-field electroretinography (ERG) findings of six 41–71 year old independent patients complaining of night blindness, Tsunoda et al. found that the peripheral FAF patterns in these patients closely resemble autofluorescence in trickling AMD.

With the hope of providing an overview of peripheral lesions in UWF studies of the retina, a 2019 meta-analysis by Forshaw et al. systematically reviewed all studies using ultra-widefield imagining in AMD eyes [31]. Of the twelve eligible clinic-based studies of 3261 or more eyes in total, peripheral lesions (RPE change, retinal atrophy, drusen) were found in 82.7% of AMD eyes, compared to about 33.3% of healthy eyes. In order to facilitate a more systematic comparison of UWF images, studies that utilized similar grid analysis techniques were chosen [31]. The meta-analysis successfully confirmed that AMD is indeed a pan-retinal disease. Without the aid of a more standardized and detailed method of categorizing peripheral retinal lesions, such meta-analyses are limited in their ability to determine possible patterns of peripheral retinal change across a wide array of publications. For this reason, future studies must employ a standardized retinal grid protocol such as the one presented in our study.

## 8. Conclusions

Ultra-widefield imaging has become an important cornerstone upon which we can study peripheral retinal findings more systematically. Within the past two decades, a variety of new UWF imaging modalities have given us an understanding of extramacular manifestations in AMD. These advancements have paved the way for development of a multitude of image analysis protocols. While data analysis methods have steadily evolved over the years, there has been discordance over grid-based models of study. The plethora of approaches to grading peripheral lesions and intra-study disagreement on FAF findings has placed a limit on data accuracy and comparability. The proposed Boston grid has been developed with these limitations in mind, aiming to set a standard for UWF imaging analysis. Already, the first study utilizing the Boston grid has implicated a connection between peripheral retinal changes and AMD related visual function [28]. Nevertheless, there is still much work to be done in optimizing our study protocols. We hope that our knowledge of the retinal periphery in AMD continues to grow in concert with a greater shift to more systematic and unified analysis.
